# Antimicrobial evaluation and molecular docking studies of the combined ethanolic extract of *Mollugo cerviana* and *Mukia maderspatana*

**DOI:** 10.6026/97320630019190

**Published:** 2023-02-28

**Authors:** Sherin Rebecca F, Vidhya Lakshmi V, Jeyashree M, Elavarasi A, Ponnivedha Rajamanickam

**Affiliations:** 1 PG and Research Centre of Zoology, Jayaraj Annapackiam College for Women (Autonomous) Periyakulam, Theni, Tamilnadu, India; 2 DBT-BIF Centre, PG and Research Department of Biotechnology and Bioinformatics, Holy Cross College, Affiliated to Bharathidasan University, Tiruchirappalli-620 002, Tamil Nadu, India

**Keywords:** molecular docking, Antimicrobial, *Mollugo cerviana*, *Mukia maderspatana*

## Abstract

Medicinal plants are considered to be the source of richness in Traditional Medicine. The chosen plants Mollugocerviana (L.) and Mukiamaderas patina. (L.) are commonly used to treat various ailments in traditional medicine. In the present study these two Plants were extracted with Ethanol and were subjected to Phytochemical Analysis to confirm the presence of different Phytochemicals. After phytochemical analysis the antimicrobial efficiency of the Plant extracts were checked against different microbial pathogens. The results confirm that the combined extract of both the plants shows potent activity against selected pathogenic strains. The results clearly indicate that the activity is in a dose dependent manner which is defined as higher the concentration higher the activity. GC-MS analysis of the extracts showed the presence of Ergost-7-en-3-ol. This ligand was docked with TyRs protein of *S.aureusi* to understand the interactions and predict the affinity and the activity of the potent bioactive molecules. It shows promising interaction with respect to binding poses of interacted complex. From the current study it is proved that the chosen plants are highly loaded with nutrients and can be used as a drug target in future. Further studies are required to confirm the efficiency of the plant extracts.

##  Background:

Though microbial infections are very common throughout the world, the treatment for these infections still remains a challenge to the health care sector due to the continuous emergence of drug resistance that microbes develop against antibiotic drugs [1]. To combat this, many lines of synthetic drugs are been established to overcome the emergence of resistance. But the use of these synthetic drugs is limited due to their harmful side effects and they are very expensive [2]. This challenge can be addressed by choosing Plants as a new drug targets. The vast era of modern medication evolved originally from our ancient herbal formulations. There are many scientific evidences that prove that there are many drugs in modern medicine which are the actual analogues of Plant substances [3]. Over the past few years interest in medicinal plants has been accelerated in a great deal and numerous plant-based researches are performed to explore the best candidates for antibiotic drug targets [4]. India has been known to be a rich repository of medicinal plants and there are numerous plants derived products which proved to be a good antimicrobial agent [5]. The ability of the plants to produce the antimicrobial activity mainly relies on the presence of the secondary metabolites such as alkaloids, flavonoids, steroids and Fatty acids [6]. In recent years the antimicrobial properties of different herbal plants are being continuously reported worldwide [7]. In the field of Plant research, Polyherbal formulation and synergistic activity between the plants has gained a greater attention due to the combined metabolic behaviour of the potent compounds in the plants. It is also evident that in polyherbal medicine lower doses are always needed to achieve the desire pharmacological actions, thus reducing the risk of negative side effects in the human body [8]. In certain cases of multiple complications, the combination of various herbs simultaneously acts on different targets to give maximum effects [9]. All of these positive characters promote polyherbal formulation as an alternative strategy to reduce the use of synthetic antimicrobial drugs and this paves the way to the new era of herbal medicine Mollugocerviana (L.) is a short annual herb commonly and widely distributed in the south Indian states. The leaves and the roots of the plants are traditionally used as medicines in most of the villages due to its various medicinal properties [10]. This plant has been proved to be a good hepatoprotective agent, and also this plant has gained more importance as they have a good anti - inflammatory, antioxidant and antimicrobial properties. Decoctions of roots and leaves are being used to treat gout and rheumatism [11]. *Mukiamaderaspatina*. (L.) is a climber found vastly distributed in tropical parts of south India and Sri Lanka [12]. This plant is loaded with rich phytochemicals and the leaves of the plant have been traditionally used in the interior parts of South India in treating Asthma, bronchitis, and also as a diuretic [13]. In ethnomedicine the paste of the crushed plant seeds is used as pain relief medicine and also to treat tooth aches. The ayurvedic system of medicine uses this plant as an expectorant and carminative agent [14]. Therefore, it is of interest to evaluate the antimicrobial activity of the ethanolic extract of *Mollugo cerviana* and Mukia maderaspatina against the selected microorganisms of bacterial and fungal pathogens.

## Materials and Methods:

## Collection of plant materials:

The whole plants of Mollugocerviana and Mukia maderaspatina were carefully collected from the hilly areas of Western Ghats in Theni district, Tamil Nadu. The healthy plants were carefully chosen, thoroughly washed with distilled water an air dried at room temperature

## Microbial strains:

All the standard microorganisms for the present study were obtained from MTCC, Chandigarh. The microbial strains used in this study include three bacteria and four fungi. This includes *Staphylococcus aureus*, *Bacillus subtilis*, *Streptococcus pyogenes* and the fungal strains includes *Aspergillus niger*, *Candida albicans*, *Cryptococcus neoformans* and Sporothrix *schenckii*. All these microbial cultures were suspended and maintained in Nutrient broth (Himedia) at 37°deg;C and on Agar Plates (Himedia) at 4°C.

## Plant extracts preparation:

The plant materials were finely pulverized into powder. About five grams of each powdered plant material were weighed and mixed together. To this mixture 50ml of ethanol is added and soaked for 72 hrs with periodic stirring and this mixture was extracted through Soxhlet apparatus and the solvent was removed through rotary vacuum evaporator. Finally, the precipitate was collected and diluted appropriately and used for further analysis.

## Screening for antimicrobial activity:

Disc Diffusion method proposed by National Committee for clinical Laboratory Standards (NCCLS) [15] was used to check the antimicrobial activity of the medicinal plants

## Antibacterial activity:

To check the efficiency of the antibacterial activity, Petri plates containing 20 ml Nutrient agar medium were seeded with 24hr culture of bacterial strains and this was standardized with 0.5% MacFarland solution at 550nm.Four wells of 8mm diameter and 3mm depth were made on the agar plate and the plant extracts of varying concentrations (500, 250,100 and 50 µg/ml) were carefully added to the wells. The Plates were incubated for 24hrs at 37 ° C and the results were tabulated. Antibiotic Standard (10 µg) was used as a positive control. Antibacterial activity was determined by measuring the zone of inhibition (mm). The values were calculated using Graph Pad Prism 6.0 software (USA).

## Antifungal activity:

To analyse the antifungal activity Petripaltes containing 20 ml of Potato dextrose agar medium was seeded with 72 hr culture of fungal strains, wells were made and different concentration of the plant extract (500, 250,100 and 50 µg/ml) were added, these were then incubated at 28 ° C for 72 hrs. The standard Antibiotic drug (10 µg) was used as appositive control. The antifungal activity was assayed by calculating the zone of inhibition (mm) and the values were measured using Graph Pad Prism 6.0 software (USA).

## GC-MS analysis:

The GC-MS analysis was performed on a combined GC-MS instrument (ITQ 900 Model of Thermos Fisher Scientific make) using a HP-5 fused silica gel capillary column. The method to perform the analysis was designed for both GC and MS using the XCaliber Software provided with the machine. A 1 µl-aliquot of sample was injected into the column using a PTV injector whose temperature was set at 275° C. The GC program was initiated by a column temperature set at 60°C for 5 min, increased to 300 ° Cat a rate of 8 C/min, held for 10 min. Helium was used as the carrier gas (1.5 ml/min). The mass spectrometer was operated in EI mode with mass source was set at 200°C. The chromatogram and spectrum of the peaks were visualized using Qual Browser software. The particular compounds present in the samples were identified by matching their mass spectral fragmentation patterns of the respective peaks in the chromatogram with those stored in the National Institute of Standards and Technology Mass Spectral database (NIST-MS, 1998) library.

## Molecular docking studies:

The three dimensional structure of the ligands obtained from GC/MS chromatogram were downloaded from pubchem database. The structures of all the compound was energy minimized by with Merck Molecular Force Field (mmff94) through Open Babel 2.3.0. The ligand was then converted into PDBQT format using PyRx v.0.8 [16].

## Protein preparation:

The Crystal structure of protein (PDB ID: 1jij) was downloaded from PDB database. The cocrytallized ligand and water molecules were removed from the protein. Polar hydrogens were added with AutoDockTools1.5.6 (ADT). The protein structure was then converted into PDBQT format using PyRx v.0.8.

## Molecular docking:

The procedure of docking of ligands with the receptor has been performed using Autodock version 0.8 of pyrx software. A grid box was created to reside the entire protein inside the box for blind docking. The ligands were chosen based on the lowest binding affinity, which has a value of Root Mean Square Deviation (RMSD) ≤1.0 Å, the protein-ligand interactions were visualized using the Discovery Studio Visualizer 2019 [17].

## Results and Discussion:

**Figure 2:**
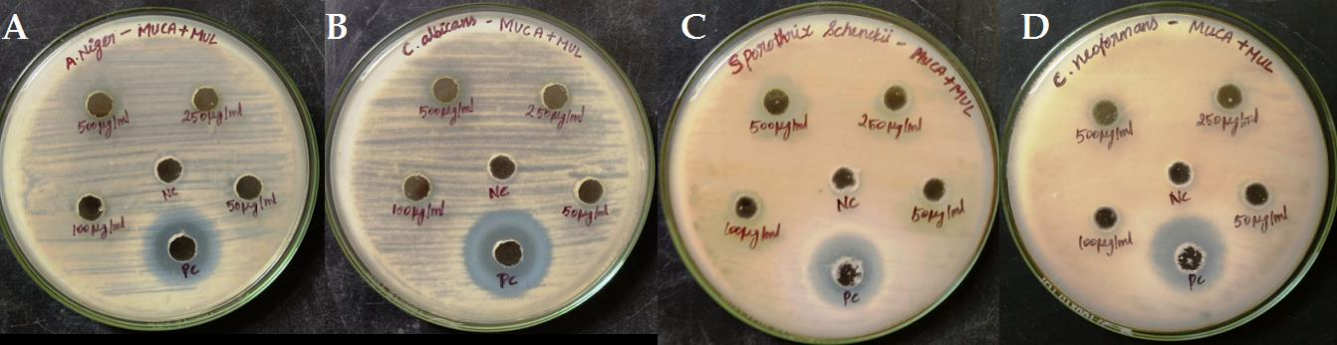
Antifungal activity of the polyherbal extract against fungal Pathogens. Antifungal activity is expressed as zone of inhibition in mm (A-*Aspergillus niger*, B-*Candida albicans* C-Sporothrix *schenckii* D-*Cryptococcus neoformans* ).

In the current study the antimicrobial activity of the combined Plant extracts of *Mollugo cerviana* and Mukia maderaspatina prepared in ethanol were tested for its antimicrobial efficiency against the selected bacterial strains of *Staphylococcus aureus*, *Bacillus subtilis* and *Streptococcus pyogenes* and fungal strains of *Candida albicans*, *Aspergillus niger*, *Cryptococcus neoformans* and Sporothrix *schenckii*. The result findings tabulated in Table 1 and Table 2 gives a good insight that the combined extract has good antimicrobial potential against all tested microorganisms. The polyherbal extract shows highest activity against *Bacillus subtilis* with the zone of inhibition of about 21.5±0.7 mm. This formulation also possesses a good activity against *Staphylococcus aureus* with the zone of inhibition 16.5±0.7 mm which is followed by *E.coli* with 15.5± plusmn;0.7 mm.(Similarly, the extract has a good potential against the fungal strains of *Cryptococcus neoformans* and *Aspergillus niger* with the zone of inhibition of around 10.25±0.35mm. It produced a zone of 5.5±0.7mm against Sporothrix *schenckii* in the higher concentration of 500 µg/ml. (Figure 2) This formulation was highly effective against the bacterial strains when compared with the fungal strains. Earlier studies reveals that the plant extracts of *Mollugo cerviana* was effective against many gram positive and negative bacteria with the zone of inhibition of around 12mm to 30 mm [18] and the extracts of Mukia maderaspatina was highly effective against the bacterial strains with the inhibition zone ranging from 16mm to 22mm [19]. This gives clear evidence that the combination of these two extracts has a very good potent activity against the bacterial strains when compared with the individual extracts.

##  Molecular docking studies:

**Figure 3:**
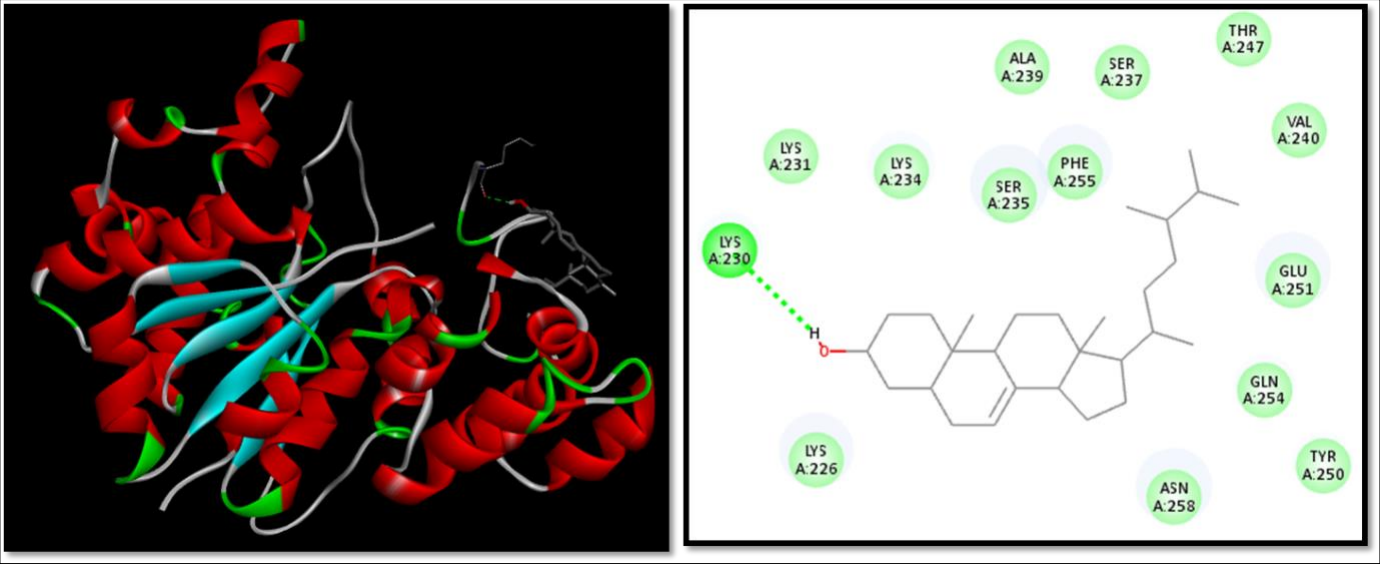
Interaction between TyRs protein and Ergost-7-en-3-ol

Molecular docking studies were carried out for all the compounds obtained through GC.MS results (Table 3). Compound Ergost-7-en-3-ol showed high binding affinity score with TyRs of *S.aureusi*. Binding affinity score helps to understand the interactions and predict the affinity of the potent bioactive molecules. The docking results are monitored by scoring functions that predict how well the ligand binds in a particular docked pose. The performance of a scoring function possibly depends on binding characteristics present in a particular protein-ligand interface. In the present study hydrogen bonds interactions contribute to the final score of a particular protein-ligand interaction (Table 4). Ergost-7-en-3-ol showed hydrogen bond interaction with LEU230 (Figure 3). These interactions indicate that this compound can bind strongly to TyRs of S. *aureusi* and may potentially produce inhibitory effects against staphylococcus infection.

## Conclusion:

*Mollugo cerviana* and *Mukia maderspatana* are the most common medicinal plants that are frequently used in the interior rural areas as a traditional medicine. The present study gives a clear picture that these plants represent good antimicrobial activity against the selected pathogens as they are loaded with rich phytochemicals. Till now, the pharmacological profile of these plants is not been documented, extensive research is essential to study the detailed biological properties of these plants. The results of the present study reveals that the combined extract of the two plants shows the presence of the compound Ergost-7-en-3-ol, and when this docked with TyRs protein of *S.aureusi*, the results shows that it has a strong binding affinity against the bacteria. This study gives a conclusion that this compound serves as a very good inhibitory agent against staphylococcus infection. Further *in vivo* studies are required to check the safety and efficacy of the Plant extracts.

## Figures and Tables

**Figure 1:**
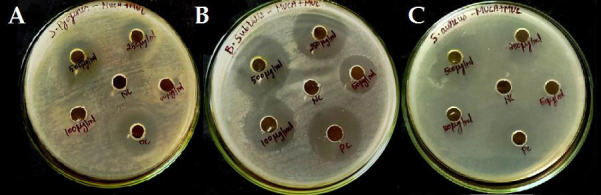
Antibacterial activity of the polyherbal extract against bacterial Pathogens. Antibacterial activity is expressed as zone of inhibition in mm (A-*Streptococcus Pyrogens*, B-*Staphylococcus aureus* C-*Bacillus subtilis*).

**Table 1:** Antibacterial activity of the combined extracts of *Mollugo cerviana* and *Mukiamaderaspatina* against tested microorganisms SD represents mean value Standard Deviation

**S. No**	**Name of the test organism**		**Zone of inhibition (mm)**			
				SD± Mean		
		500µg/ml	250µg/ml	100µg/ml	50µg/ml	PC
1	*Staphylococcus aureus*	16.5±0.7	15.25±0.35	0	0	17.5±0.7
2	*Bacillus subtilis*	21.5±0.7	19.25±0.35	18.25±0.35	10.25±0.35	19.5±0.7
3	*Streptococcus pyogenes*	15.5±0.7	13.25±0.35	12.25±0.35	10.25±0.35	16.5±0.7

**Table 2:** Antifungal activity of the combined extracts of *Mollugo cerviana* and *Mukiamaderaspatina* against tested Fungal Pathogens SD represents mean value Standard Deviation

**S.NO**	**Name of the test organism**		**Zone of inhibition (mm)**			
				SD ± Mean		
		500 µg/ml	250 µg/ml	100 µg/ml	50 µg/ml	PC
1	Sporothrix *schenckii*	5.5±0.7	4.25 ±0.35	0	0	13.75±1.06
2	*Cryptococcus neoformans*	10.25±0.35	5.25±0.35	0	0	14.5±0.7
3	*Candida albicans*	9.5±0.7	0	0	0	15.25±0.35
4	*Aspergillus niger*	10.25±0.35	7.25±0.35	0	0	14.5±0.7

**Table 3:** GC/Ms analysis

**Name**	**Retention time**	**Area %**
1,2-Benzenedicarboxylic Acid, Diethyl Ester	19.97	1.19
2-Hexadecen-1-Ol, 3,7,11,15-Tetramethyl-, [R-[R*,R*-(E)]]-	28.98	23.55
9,12,15-Octadecatrienoic Acid, (Z,Z,Z)-	29.376	2.03
Linoleic Acid Ethyl Ester	29.731	6.74
Ethyl (9z,12z)-9,12-Octadecadienoate #	29.822	22.37
Octadecanoic Acid, 17-Methyl-, Methyl Ester	30.234	2.88
Hexadecanoic Acid, 2-Hydroxy-1-(Hydroxymethyl)Ethyl Ester	34.586	3.96
Dioctyl Phthalate	34.877	2.92
9,12,15-Octadecatrienoic Acid, Ethyl Ester, (Z,Z,Z)-	36.916	1.27
Octadecanoic Acid, 2,3-Dihydroxypropyl Ester	37.181	1.26
Squalene	38.33	1.88
Silikonfett	38.385	0.53
Silicone Oil	39.015	2.04
(+)-.Gamma.-Tocopherol, O-Methyl-	39.06	2.44
1,4-Benzenediol, 2,5-Bis(1,1-Dimethylethyl)-	39.105	1.08
Silicone Grease, Siliconfett	39.135	0.61
Heptasiloxane, Hexadecamethyl-	39.19	0.75
Trimethyl[2-(Trimethylsilyl)Phenyl]Silane	39.37	1.96
Ergost-7-En-3-Ol	39.825	1.79

**Table 4:** Interaction between TyRs protein and Ergost-7-en-3-ol

**S.NO**	**Protein**	**Ligand**	**Binding Affinity**
1	TyrRS (1JIJ)	Ergost-7-en-3-ol	-7.3
